# Early Autonomic Dysfunction in Traumatic Brain Injury: An Article Review on the Impact on Multiple Organ Dysfunction

**DOI:** 10.3390/jcm14020557

**Published:** 2025-01-16

**Authors:** Pattrapun Wongsripuemtet, Tetsu Ohnuma, Zeljka Minic, Monica S. Vavilala, Joseph B. Miller, Daniel T. Laskowitz, William J. Meurer, Xiao Hu, Frederick K. Korley, Huaxin Sheng, Vijay Krishnamoorthy

**Affiliations:** 1 Critical Care and Perioperative Population Health Research (CAPER) Program, Department of Anesthesiology, Duke University, Durham, NC 27708, USA; tetsu.ohnuma@duke.edu (T.O.); vijay.krishnamoorthy@duke.edu (V.K.); 2 Department of Anesthesiology, Faculty of Medicine, Siriraj Hospital, Mahidol University, Bangkok 10700, Thailand; 3 Departments of Anesthesiology, Duke University, Durham, NC 27708, USA; huaxin.sheng@duke.edu; 4 Department of Emergency Medicine, Wayne State University School of Medicine, Detroit, MI 48202, USA; zminic@med.wayne.edu; 5 Faculty of Biotechnology and Drug Development, University of Rijeka, 51000 Rijeka, Croatia; 6 Departments of Anesthesiology and Pain Medicine, University of Washington, Seattle, WA 98109, USA; vavilala@uw.edu; 7 Department of Emergency Medicine, Henry Ford Health System, Detroit, MI 48202, USA; 8 Department of Neurology, Duke University, Durham, NC 27708, USA; daniel.laskowitz@duke.edu; 9 Department of Emergency Medicine, University of Michigan, Ann Arbor, MI 48109, USA; wmeurer@med.umich.edu (W.J.M.); korley@med.umich.edu (F.K.K.); 10 Department of Emergency Neurology, University of Michigan, Ann Arbor, MI 48109, USA; 11 School of Nursing, Emory University, Atlanta, GA 30322, USA; xiao.hu@emory.edu; 12 The Max Harry Weil Institute for Critical Care Research and Innovation, Ann Arbor, MI 48109, USA

**Keywords:** traumatic brain injury, autonomic dysfunction, multiorgan dysfunction

## Abstract

**Background/Objectives:** Traumatic brain injury (TBI) is a complex condition and a leading cause of injury-related disability and death, with significant impacts on patient outcomes. Extracranial organ involvement plays a critical role in the outcome of patients following TBI. **Method:** This review aims to provide a comprehensive overview of the pathophysiology, clinical presentation, and challenges in diagnosing patients with autonomic dysfunction after TBI. The databases used in this review include PubMed/MEDLINE, Cochrane Central Register, and Scopus. **Results:** Of 172 articles identified for screening, 98 were ultimately included in the review. **Conclusion:** This review summarized the current evidence on the pathophysiology, clinical presentation, and diagnosis of early autonomic dysfunction. It also emphasizes the effects of autonomic dysfunction on end-organ damage. These insights aim to guide clinicians and researchers toward improving the care for and understanding of autonomic dysfunction in TBI patients, while underscoring the need for further research in this area.

## 1. Introduction

Traumatic brain injury (TBI) is a heterogeneous condition, and moderate to severe TBI is a primary cause of injury-related disability and death [1]. In 2016, there were more than 27 million new cases of TBI globally, 2.5 million emergency department visits, and 56,800 TBI-related deaths in the US during 2014 [2]. TBI not only leads to mortality but also results in years lived with disability [3]. In 2016, the overall healthcare costs attributable to non-fatal TBI in the US were 40.6 billion [4].

In TBI, primary and secondary brain injuries play important roles in the overall impact on cognitive function, physical function, and patient outcomes. The primary injury refers to the direct insult from the external mechanical force that causes injury, which is not alterable once it occurs and initiates the cascade of complex systemic alterations. Secondary injury of the brain occurs as a complication after the initial injury that includes ischemic and hypoxic damage, cerebral edema, increased intracranial pressure, hydrocephalus, infection, and other local and systemic cascades that can further harm the brain tissue [5].

The systematic effects of brain injury manifest as extracranial multiorgan dysfunction (MODS), occurring in over 68% of moderate to severe TBI patients within the first 72 h after admission [6]. Dysfunction in the extracranial organs can create a vicious cycle, exacerbating further brain damage. Autonomic dysfunction has been identified as a significant contributor to MODS development following TBI, especially in severe TBI [7]. This dysfunction precipitates a cascade of physiological disruptions, resulting in dysfunction across multiple extracranial organ systems, including the pulmonary, circulatory, renal, and coagulation systems. Consequently, TBI patients who develop MODS experience a poor-long term prognosis, which is associated with increased mortality rates [8,9,10]. Yet the importance of the prevention and treatment of early autonomic dysfunction could help improve patient outcomes, from initial survival to their long-term outcomes. The purpose of this scoping review is to present evidence of the pathophysiology of and diagnosis of early autonomic dysfunction and its impact on extracranial organ dysfunction in terms of improving autonomic balance and preventing extracranial organ failure following moderate to severe TBI.

## 2. Search Strategies

The search strategies used in this review involved the following databases: PubMed/MEDLINE, Cochrane Central Register, and Scopus. The articles were selected using the following MeSH terms: dysautonomia, autonomic storms, sympathetic storms, paroxysmal sympathetic storm, autonomic seizure, hypothalamic storms, early autonomic dysfunction, TBI, diencephalic epilepsy, midbrain dysregulatory syndrome, paroxysmal autonomic instability with dystonia (PAID), multiorgan dysfunctions, and traumatic brain injury (TBI).

The inclusion criteria focused on studies published between 1990 and 2024 that addressed the pathophysiology, diagnostic criteria, management, and outcomes in patients with these dysfunctions. The review was conducted by three authors (P.W., T.O. and V.K.), who noted significant variability in the diagnosis and terminology related to autonomic dysfunction.

### 2.1. Results

This narrative review identified 172 articles. After removing duplicate articles, studies with irrelevant populations or outcomes or publication dates outside of our criteria, literature not in the English language, and others outside the inclusion range, 98 articles were ultimately included in the review.

### 2.2. The Epidemiology and Definition of Early Autonomic Dysfunction in TBI

Early autonomic dysfunction is a common occurrence after severe TBI, with an incidence ranging from 8 to 33% [11,12,13]. There is a correlation between the severity of the initial TBI and autonomic dysfunction [14], which commonly occurs during the stage of recovery or “coma emergence”.

Autonomic dysfunction is associated with increased morbidity and worse clinical outcomes [15], but it is often overlooked due to the lack of standardized terminology, which remains a major barrier to the proper diagnosis, treatment, and accurate determination of its true incidence. Various terms have been used to describe this condition [16]. Early autonomic dysfunction is a syndrome caused by disproportionate pathological sympathetic activation, characterized by a severe paroxysmal increase in blood pressure, heart rate (HR), respiratory rate (RR), temperature, sweating, muscle spasticity, and posturing [17]. It usually develops in moderate to severe TBI [18].

### 2.3. Pathophysiology

The pathophysiology of autonomic dysfunction after TBI involves the dysregulation of the autonomic nervous system (ANS) due to injury to various parts of the brain. Areas such as the hypothalamus, the nucleus tractus solitarius, and the medulla play crucial roles in controlling and regulating the ANS [19]. Its pathophysiology is not fully understood, but it is considered to be a disconnection phenomenon. This involves paroxysms triggered by a loss of inhibitory control over the excitatory autonomic centers, including the paraventricular hypothalamic nucleus, the lateral periaqueductal gray substance, the lateral parabrachial nucleus, or the rostral ventricular medulla [20]. The ANS extends beyond its role in the vascular system, as it also innervates the cardiac muscle, smooth muscle, and various endocrine and exocrine glands, thereby influencing most of the tissues and organs throughout the body. The ANS exerts control over blood pressure, gastrointestinal responses, thermoregulation, renal perfusion, contraction of the urinary bladder, and focusing of the eyes [21].

Although there is debate regarding the pathophysiology of autonomic dysfunction, it is widely agreed that this condition is primarily driven by the loss of inhibition of the excitatory sympathetic neurons, without parasympathetic involvement [22]. It is important to consider that the parasympathetic response, which should counteract sympathetic activity, is also nonfunctional. Parasympathetic activity is mainly regulated by the orbitofrontal cortex, which normally inhibits sympathetic activity. If this area is damaged, this leads to increased adrenomedullary catecholamines during autonomic dysfunction, and the negative feedback loop fails to function properly.

Following TBI, the activation of the hypothalamic–pituitary–adrenal axis results in an uncontrolled surge of adrenergic energy. This leads to the release of large amounts of catecholamines, including epinephrine, norepinephrine, and dopamine. These catecholamines directly impact the end organs and also play a role in the inflammatory response through the release of cytokines. It directly affects the function of a wide range of organs, including the heart, lungs, kidneys, and lymphoid organs. Dysfunction of the autonomic nervous system, in general, has been found to induce abnormalities in organ systems throughout the body [23,24]. Disruption of the blood–brain barrier (BBB) during TBI leads to the release of brain-derived antigens such as glial fibrillary acidic protein, S100, and myelin basic protein into the systemic circulation. This release triggers the production of cytokines and chemokines, including tumor necrosis factor-α, IL-6, and IL-1β, which leads to a systemic inflammatory response [25].

In patients with autonomic dysfunction, blood pressure fluctuations may be observed as early as on the second day after injury. Plasma catecholamine levels are correlated with these fluctuations [26] and also with the severity of the injury, predicting both functional outcomes and mortality [27,28,29]. Previous studies have indicated that autonomic dysfunction serves as more than just an independent predictor of increased morbidity, mortality, or a greater proportion of unfavorable outcomes [8,9,10]. It also correlates with prolonged hospital stays and lower Disability Rating Scale (DRS) scores at discharge [30].

### 2.4. Diagnostic Criteria

Autonomic dysfunction during the acute phase of TBI commonly presents as paroxysmal sympathetic hyperactivity (PSH), a term introduced by Rubinstein in 2007 [13]. PSH is characterized by episodic increases in vital signs, such as heart rate, blood pressure, respiratory rate, and temperature, along with abnormal, non-purposeful movements, hypertonia, or spasticity. Various terms have been used to describe this condition, including autonomic dysfunction syndrome, dysautonomia, and paroxysmal autonomic instability with dystonia, leading to diverse diagnosis criteria and confusion in the search terms for this condition. However, all of these names refer to the same underlying condition of ANS dysregulation [13,31]. Subsequently, in 2014, a conceptual clinical definition and diagnostic criteria for this syndrome were established [18]. It is defined clinically as a syndrome observed in survivors of a severe acquired brain injury, characterized by simultaneous, paroxysmal, transient increases in sympathetic (elevated heart rate, blood pressure, respiratory rate, temperature, and sweating) and motor (posturing) activity.

The diagnosis of PSH has a specific scoring system that can be applied in clinical settings. The diagnostic criteria are still challenging as there is no specific test for it. The challenge in diagnosing it during the acute or subacute phase lies in the fact that patients with a traumatic brain injury (TBI) typically receive sedation to minimize the risk of further secondary brain injury. In most cases, a diagnosis is made according to exclusion. The 2014 consensus established uniform clinical diagnosis criteria for paroxysmal sympathetic activity by using the Paroxysmal Sympathetic Hyperactivity Assessment Measure (PSH-AM) scale is by far the best diagnostic tool for PSH in TBI patients [15,18]. The PSH-AM comprises two main constructs, (a) a Clinical Feature Severity (CFS) score and (b) a Diagnosis Likelihood Tool (DLT) score, and combining these domains results in the PSH-AM score (Table 1). This scoring system helps in assessing the severity of the clinical features associated with autonomic dysfunction. However, the scoring system may offer late detection and render a diagnosis difficult to make in an ICU setting, including the difficulty quantifying the severity of sweating and posturing during an episode.

While autonomic dysfunction manifests clinically with the above criteria, its biological manifestations occur much earlier following severe TBI. Several techniques are employed to measure and describe the complex system of autonomic dysfunction. One such method is the analysis of heart rate variability (HRV); a loss in HRV reflects sympathovagal imbalance [32]. Reduced variability in the R-R series in an electrocardiogram (EKG) reflects dysregulation of the ANS and the sinoatrial (SA) node [33]. Various studies have demonstrated a correlation between low HRV measures, the severity of the illness, and poor outcomes [14,34,35].

Heart rate variability (HRV) is a non-invasive marker derived from electrocardiography that reflects the influence of both the sympathetic and parasympathetic components of the ANS on the heart’s sinus node. Studies have shown a correlation between HRV and TBI, with HRV serving as a predictor of poor outcomes [9,36]. HRV measures the variation in both the instantaneous heart rate and the RR intervals (the time between successive QRS complexes during normal sinus depolarization), providing insights into baseline autonomic regulation. HRV can be measured over periods ranging from a few minutes (0.5 to 5 min) to 24 h using a Holter monitor. The two primary methods of analysis are the “time domain” (which evaluates the RR intervals of successive heartbeats over time) and the “frequency domain” (which analyzes the spectrum of the heart rate’s oscillatory components). A common statistical analysis is the standard deviation of the normal-to-normal interval (SDNN), which assesses the variability in the R-to-R intervals [37]. The frequency-domain analysis divides HRV into “low-frequency” (LF) components, which are associated with sympathetic activity, and “high-frequency” (HF) components, which are linked to parasympathetic activity. The ratio of LF to HF components is often considered an important indicator of autotomic balance. A lower “LF/FH” ratio typically indicates parasympathetic dominance, whereas a higher “LF/HF” ratio suggests sympathetic dominance [38].

The arterial pulse wave (APW) has a distinct morphology and contour that can be used as an indirect method to evaluate the sympathetic response through arterial stiffness as a result of the sympathetic nervous system. This response not only affects arterial blood pressure and heart rate but also induces changes in the APW contour. These changes can be observed through characteristics such as the systolic slope and the stiffness index [39].

Cardiac baroreceptor sensitivity (BRS) is another important metric for evaluating ANS impairment. It correlates an increased ICP with a higher BRS, while a low average BRS is associated with a greater risk of a poor prognosis [40]. BRS measures the responsiveness of the baroreceptors to the change in blood pressure. Alterations in the baroreceptor reflex contribute to the imbalance between the sympathetic and parasympathetic activity. BRS can be measured using direct or indirect stimulation of the arterial baroreceptors, such as through the Valsalva maneuver or vasoactive drug administration. This method evaluates the relationship between changes in RR intervals and an increase in systolic arterial pressure [41,42].

A diagnosis of autonomic dysfunction in TBI often requires ruling out other conditions, as its underlying pathophysiology remains unclear and may overlap with that of other injuries or medical issues. Its clinical presentation can mimic various severe illnesses. For instance, autonomic hyperactivity is observed in conditions such as neuroleptic malignant syndrome, malignant hyperthermia, serotonin syndrome, and thyroid storm [43]. Symptoms such as tachycardia may also occur with pain, pulmonary embolism, or septicemia. Furthermore, posturing episodes associated with autonomic dysfunction could be misinterpreted as epileptic seizures [44].

### 2.5. Clinical Settings

Autonomic dysfunction symptoms can appear at any stage of the TBI course, ranging from the intensive care unit to the rehabilitation phase, with various terminologies, such as dysautonomia, sympathetic hyperactivity, or paroxysmal sympathetic activity, commonly used to describe the acute phase of autonomic dysfunction in the early stages of TBI [45]. During the early phase of TBI, patients are often sedated, and the classical feature of autonomic dysfunction may be masked [41]. However, the symptoms of autonomic dysfunction typically develop within the first week post-TBI, with an average onset of 5.9 days [12]. Data published in 2023 show that the temporal patterns of baroreflex sensitivity, which reflect autonomic dysfunction following traumatic brain injury, peak approximately 2 days after a TBI [40]. The pattern of the variation in heart rate was reported to be a strong signal on days 2–4 for a non-survival TBI group, while in the survival group, it occurred at a later stage, on days 8–13. This observation suggests that exhibiting a strong signal may reflect a higher risk of poor outcomes [10]. Studies have shown evidence of clinically relevant autonomic dysfunction persisting into the chronic stage after moderate to severe TBI. Patients among those who survive can be in the chronic phase of injury up until as late as 6–299 months [45].

The presentation symptoms of autonomic dysfunction have multiple signs, such as hypertension, tachycardia, tachypnea, hyperthermia, hyperhidrosis, abnormal posturing, spasticity, and increased intracranial pressure [22,46]. The symptoms that manifest first are often hypertension and tachycardia [17,41]. These symptoms can occur spontaneously or are often triggered by various stimulations, such as pain, stimulation of body movements, suctioning, or bladder distension [31].

Although the symptoms predominantly exhibit a sympathetic pattern in the acute phase, q study by Lucia Li reported a broad range of autonomic dysfunction symptoms in the post-acute period after moderate to severe TBI. These symptoms can span the entire spectrum of autonomic dysfunction. Patients may experience parasympathetic, sympathetic, or mixed dysfunction following a TBI. This study emphasizes the diverse and complex nature of autonomic dysfunction in TBI patients [45].

### 2.6. The Impact of Autonomic Dysfunction on Organ Dysfunction

- *The cardiovascular system*

The cardiac abnormalities following brain injury, particularly after a subarachnoid hemorrhage, are well characterized, including global myocardial dysfunction, observed in echocardiography and electrocardiographic changes [47,48]. The theory of an elevated sympathetic tone and a surge of catecholamines has been proposed, leading to cardiac hypertrophy or myocardial ischemia [25,49]. The sympathetic activation causes vasoconstriction, tachycardia, and hypertension, resulting in increased oxygen demand and ultimately leading to subendocardial ischemia and ventricular dysfunction.

A retrospective study conducted by Prathep et al. revealed significant insights. Among patients with an isolated TBI, 22.3% exhibited abnormal echocardiograms, with 12% having a reduced Left Ventricular Ejection Fraction (LVEF) and 17.5% presenting with a new regional wall motion abnormality (RWMA). A total of 30.6% of patients had elevated CK-MB levels, and 43.3% of this group exhibited abnormal echocardiography findings [50]. In their study, abnormal echocardiograms were independently associated with all-cause in-hospital mortality.

Similarly, a prospective study by Krisnamoorthy et al. found that 22% of patients with a moderate to severe TBI experienced systolic dysfunction, whereas none exhibited such dysfunction in cases of a mild traumatic brain injury [51]. A higher severity of TBI seems to contribute to the larger effect in abnormal ECG changes. The study by Hasanin et al. found abnormal ECG findings in 62% and abnormal echocardiographic findings in 28% of the severe TBI group, but this was unrelated to mortality risk factors after their multivariate analysis [52].

A TBI manifests as a variety of ECG changes. Changes such as prolongation of the QT interval, ST segment abnormalities, flat or inverted T waves, U waves, peaked T waves, Q waves, widened QRS complexes, and tachycardia have been described [53,54]. Ischemic-like ECG changes in TBI may occur even without myocardial infarction and are related to intracranial hypertension. Additionally, non-ischemic ECG changes, repolarization abnormalities such as a prolonged QT interval, and end-repolarization abnormalities are observed in cases of an isolated TBI and are associated with cardiac dysfunction [54]. One study reported cardiac dysfunction in 13% of patients with a moderate to severe TBI, with a higher proportion of ECG abnormalities in the group that developed dysfunction [55]. In studying heart rate variability, it has been found to be linked to the mortality rate. Papaioannou et al. showed that mortality in acute brain injury patients is linked with decreased heart rate variability, low baroreflex sensitivity, and a sustained decrease in low-frequency/high-frequency heart rate signals [10]. Sykora et al. emphasized the association of low baroreceptor reflex sensitivity and a high-frequency band of heart rate variability with mortality [9]. Despite ECG and echocardiographic changes, no interventions were required [56], and abnormalities were usually reversible within the first week of hospitalization [51]. Following a TBI, the initial catecholamine response is characterized by hyperdynamic circulation, featuring tachycardia and hypertension. However, it is important to recognize that marked sinus bradycardia, known as the Cushing response, may also occur due to the baroreceptor reflex activity triggered by elevated intracranial pressure (ICP), resulting from various factors, such as direct injury, cerebral swelling, and diffuse axonal injury.

- *The respiratory system*

Acute lung injury (ALI) occurs in approximately 22% of TBI patients and significantly elevates the mortality rates in this population [57,58]. Various mechanisms contribute to the development of lung injury, including a systemic inflammatory response, induced arterial hypertension to maintain cerebral perfusion pressure, and neurogenic pulmonary edema [59,60,61]. Neurogenic pulmonary edema is a form of Acute Respiratory Distress Syndrome (ARDS) triggered by severe central nervous system injury and exhibits an incidence ranging from 2% to 32% in cases of neurologic insult [62,63,64,65]. The onset of ARDS can be acute, occurring within 30 to 60 min after injury, or delayed, occurring within 12 to 72 h in most patients. Typically, spontaneous resolution of NPE occurs within 72 h [66,67,68].

It is characterized by the extravasation of fluid from the blood into the alveolar and interstitial space in the lungs, triggered by the sympathetic overactivity which results from a sudden increase in intracranial hypertension [61,63,69,70,71,72]. The severity of lung injury is associated with the severity of TBI and correlates with increased mortality after TBI [73].

ARDS manifests clinically as dyspnea, tachypnea, tachycardia, and cyanosis, accompanied by abnormal lung sounds, such as rales, crackles, or rhonchi [67]. Radiographic findings typically reveal diffuse bilateral alveolar opacities without cardiomegaly. The management of ARDS primarily focuses on treating the underlying neurologic condition to suppress the sympathetic discharge responsible for lung injury. Timely recognition, lung-protective ventilation strategies, and the use of diuretics are crucial in the symptomatic approach. Although the use of anti-α-adrenergic agents has shown promising results in animal models [74], one case report described a rise and fall in catecholamine levels following treatment [75]. A lack of human trials on their use raises the question of their safety and efficacy. Stellate ganglion block, proposed for complete sympathetic denervation, may offer potential benefits, but to date, no published data support its real benefit in this population [76]. Continued research is needed to understand and effectively manage NPE better in TBI patients.

- *Renal function*

The incidence of acute kidney injury (AKI) in traumatic brain injury (TBI) ranges from 9.2% to 23%, particularly affecting young TBI patients with normal renal function before injury. While renal replacement therapy is required by only a small fraction of patients, an association between AKI and mortality has been observed [77,78,79,80]. The comprehensive pathophysiology of AKI in TBI remains elusive, though the intricate regulation of the renal blood flow, glomerular filtration, and sodium handling by sympathetic efferent impulses from the central nervous system (CNS) is crucial. One mechanism contributing to post-TBI AKI involves an increase in sympathetic nervous system (SNS) activity, driven by the need for systolic hypertension to ensure adequate brain perfusion. Elevated SNS activation induces a catecholamine surge and activates the renin–angiotensin–aldosterone system, reducing renal glomerular perfusion and increasing renal sodium reabsorption [29]. Sustained severe hypertension may also lead to hemolysis, causing a secondary AKI due to red cell thrombus formation in the glomeruli [81].

The majority of AKI cases develop within seven days, peaking in the first three days post-injury [79]. The risk factors for AKI development in TBI include age, the severity of the illness, a low Glasgow Coma Scale (GCS) score, preinjury renal dysfunction, and insulin-dependent diabetes [78,80]. Iatrogenic factors, such as mannitol use, have been associated with an increased risk. The risk of AKI may be mitigated in patients undergoing intracranial pressure (ICP) monitoring by reducing mannitol usage [82], as suggested by the study by Robba et al., indicating an association between mannitol therapy during am intensive care unit (ICU) stay and the risk of developing AKI, presenting as a modifiable risk factor [80]. Osmotic therapies, including hypertonic saline, elevate the risk of hyperchloremia, leading to an increased chloride load in the tubular fluid and subsequent vasoconstriction of the afferent arterioles, ultimately reducing the glomerular blood flow. Other modifiable factors influencing AKI development include exposure to nephrotoxic drugs and radiocontrast agents. Understanding and addressing these factors are crucial to preventing and managing AKI in TBI patients.

- *Gastrointestinal function*

The incidence of gastrointestinal (GI) dysfunction after TBI is greater than 50%. Reports indicate a range of symptoms, including impaired swallowing, decreased pharyngeal sensation, gastric reflux, GI bleeding, delayed gastric emptying time, a decreased LES tone, gastroparesis, decreased intestinal peristalsis, and mucosal damage, with increased gut permeability [83,84,85,86,87,88,89,90,91]. The maintenance of the basic gut balance relies on intricate two-way communication via the brain–gut axis, a network consisting of various systems performing complex functions in both the gastrointestinal tract and the central nervous system. The CNS influences the gut through branches of the autonomic nervous system: the sympathetic, parasympathetic, and enteric nervous systems. In TBI, autonomic control of gut physiology is swiftly disrupted within 24 h post-injury, affecting both afferent and efferent signaling. The increased sympathetic activity after injury will initiate a stress response and contribute to GI dysmotility, including gastroparesis and food intolerance. Dysregulation of the brain–gut axis can also manifest as diarrhea [92]. The vagus nerve plays a crucial role in the intestinal mucosal response to injury and inflammation through the excitatory cholinergic reflex. Studies using vasectomized mice have demonstrated worsened injury and inflammation [93], while vagal stimulation has been shown to reduce systemic inflammation in preclinical TBI models [94,95]. TBI-induced dysautonomia and systemic inflammation can contribute to GI issues, including dysmotility and increased mucosal permeability. Impairment of mucosal barrier function was observed 4 days after injury, correlating with the disease severity and the long-term prognosis [96]. An increase in intestinal permeability can release the toxic intraluminal material and cause a progression to systemic inflammatory response syndrome (SIRS) [91,97]. The disruption of various mechanisms of normal gut physiology leads to a change in the gut microbiome environment, and the gut microbiome severs as the frontline of the immune system as an intestinal barrier. Changes include the overgrowth of the gastrointestinal microbiome, which will reduce its healthy bacterial diversity, and an increase in intestinal permeability, leading to the translocation of bacteria to the mesenteric lymph nodes and ultimately leading to bacterial infection and sepsis [98].

- *The coagulation system*

TBI-induced coagulopathy is a common and well-recognized risk in terms of poor clinical outcomes [99]. Its pathophysiology is not well understood but often involves a complex interaction between patient and injury factors. It is believed that brain-derived substrates released into the circulation after the disruption of the blood–brain barrier cause this condition. Its disruption may be caused by the primary mechanical injury or a secondary ischemic and inflammatory injury. The massive release of tissue factors can lead to hyperfibrinolysis, activation of the protein C pathway, and disseminated intravascular coagulation (DIC) [99].

Previous studies have shown that coagulopathy is equally or more prevalent in patients with an isolated TBI, even though these patients experience less blood loss and receive less fluid resuscitation, which might contribute to coagulopathy [100,101]. Dysfunction of the coagulation system can contribute to both hypo- and hypercoagulable states. The procoagulation state is evidenced by increased levels of D-dimer as early as 6 h after injury [102], and the hypocoagulable state results from the consumptive coagulopathy caused by DIC. Finally, maladaptive coagulopathy results in bleeding and the expansion of hematoma due to hypocoagulability and intravascular thrombosis due to hypercoagulability. This then leads to a secondary brain injury with both ischemic and hemorrhagic consequences [7].

**Figure 1:**
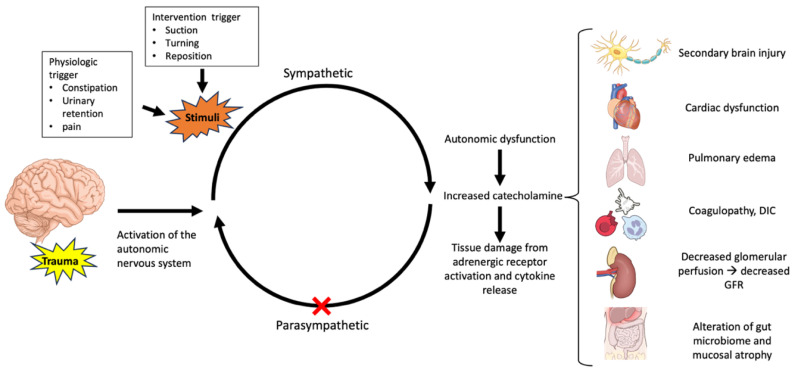
The effects of TBI on multiorgan system failure.

The effects of TBI on multiorgan system failure are summarized in Figure 1.

## 3. Conclusions and Future Directions

Autonomic dysfunction after TBI can significantly affect not only the brain but also end-organ function, ultimately impacting patient outcomes. Research and discovery are crucial for improving the diagnosis and treatment of autonomic dysfunction in TBI. This review examines the current understanding of its pathophysiology and impact on end-organ damage. Diagnosis remains challenging due to the lack of specific diagnostic tools, which can cause delays, and the treatment options are currently limited to supportive and symptomatic care. These limitations highlight the urgent need for comprehensive research to understand early autonomic dysfunction better and develop targeted interventions.

## Figures and Tables

**Table 1:** The Paroxysmal Sympathetic Hyperactivity Assessment Measure (PSH-AM) scale [15].

- Clinical Feature Scale (CFS)				
	0	1	2	3
**Heart rate**	<100	100–119	129–139	≥140
**Respiratory rate**	<18	18–23	24–29	≥30
**Systolic pressure**	<140	140–159	160–179	≥180
**Temperature**	<37.0	37.0–37.9	38.0–38.9	≥39.0
**Sweating**	No	Mild	Moderate	Severe
**Posturing during episodes**	No	Mild	Moderate	Severe
- **Diagnosis Likelihood Tool (DLT) score**				
**Characteristics**	**Presence**		**Absence**	
Antecedent acquired brain injury	1		0	
Clinical features occurring simultaneously	1		0	
Episodes are paroxysmal in nature	1		0	
Sympathetic overactivity to normally non-painful stimuli	1		0	
Absence of parasympathetic features during episodes	1		0	
Features persisting for ≥ 3 consecutive days	1		0	
Features persisting for ≥ 2 weeks post-injury	1		0	
≥2 episodes daily	1		0	
The absence of other presumed causes of features	1		0	
Feature persisting despite treatment for an alternative diagnosis	1		0	
Medication administered to decrease sympathetic features	1		0	

The interpretation of the PSH-AM score is the sum of the Clinical Feature Severity and Diagnosis Likelihood Tool scores; a PSM-AM score < 8 means PSH is unlikely, 8–16 means it is possible, and ≥17 means it is probably PSH.
